# Biomarkers in Human Anaphylaxis: A Critical Appraisal of Current Evidence and Perspectives

**DOI:** 10.3389/fimmu.2019.00494

**Published:** 2019-04-05

**Authors:** Sarah C. Beck, Thomas Wilding, Richard J. Buka, Richard L. Baretto, Aarnoud P. Huissoon, Mamidipudi T. Krishna

**Affiliations:** ^1^Department of Allergy and Immunology, Birmingham Heartlands Hospital, University Hospitals Birmingham NHS Foundation Trust, Birmingham, United Kingdom; ^2^Institute of Immunology and Immunotherapy, University of Birmingham, Birmingham, United Kingdom

**Keywords:** anaphylaxis, mast cells, tryptase, biomarkers, diagnosis

## Abstract

Anaphylaxis is a type I hypersensitivity reaction that is potentially fatal if not promptly treated. It is a clinical diagnosis, although measurement of serial serum total mast cell tryptase (MCT) is gold standard and may help differentiate anaphylaxis from its mimics. The performance characteristics of MCT assays in anaphylaxis has been variable in previous studies, due to multiple factors including differences in the definition of anaphylaxis, methods of MCT interpretation, clinical setting of anaphylaxis, causative agents, and timing of blood sample. An international consensus equation for MCT to interpret mast cell activation has been proposed and recently validated in the context of peri-operative anaphylaxis during general anesthesia. There has been an interest in the detection of newer biomarkers in anaphylaxis including platelet activation factor (PAF), chymase, carboxypeptidase A3, dipeptidyl peptidase I (DPPI), basogranulin, and CCL-2. The key determinants of an ideal biomarker in anaphylaxis are half-life, sample handling and processing requirements, and cost. There may be a role for metabolomics and systems biology in the exploration of novel biomarkers in anaphylaxis. Future studies applying these approaches might provide greater insight into factors determining severity, clinical risk stratification, identification of mast cell disorders and improving our understanding of this relatively complex acute immunological condition. Post mortem MCT evaluation is used in Forensic Medicine during autopsy for cases involving sudden death or suspected anaphylaxis. Interpretation of post mortem MCT is challenging since there is limited published evidence and the test is confounded by multiple variables largely linked to putrefaction and site of sampling. Thus, there is no international consensus on a reference range. In this state of the art review, we will focus on the practical challenges in the laboratory diagnosis of anaphylaxis and critically appraise (a) performance characteristics of MCT in anaphylaxis in different clinical scenarios (b) the role for novel biomarkers and (c) post mortem MCT and its role in fatal anaphylaxis.

## Introduction

Anaphylaxis is a systemic hypersensitivity reaction usually involving two or more organs including skin/mucus membranes, airways, cardiovascular, and/or gastrointestinal systems. It remains a clinical diagnosis and the World Allergy Organization (WAO) published diagnostic criteria based on clinical parameters (1, 2). The current gold standard laboratory test involves measurement of serum total tryptase during an acute phase followed by a baseline measurement (≥24 h) (1, 2). Whilst a rise in serum total mast cell tryptase (MCT) is diagnostic, this is not seen in all cases of anaphylaxis.

Serial MCT measurements are a useful adjunct during elective specialist assessment for investigation and long-term management of anaphylaxis. It is particularly useful in patients where there may be a paucity of historical information relating to the index episode, to differentiate anaphylaxis from its mimics or in patients with an incomplete clinical history.

As well as reviewing the basic concepts of MCT measurement and its interpretation, this article will critically appraise the published literature regarding clinical utility and performance of MCT in anaphylaxis occurring both in and outside the hospital. This article also focuses on measurement of MCT in serum obtained at post-mortem in suspected fatal anaphylaxis and explores the role for new candidate biomarkers.

## Analytical Aspects of Mast Cell Tryptase Measurement

Tryptase, a neutral serine protease, is released from secretory granules of mast cells (3–6). It exists in 2 isoforms, alpha and beta, encoded by separate genes and is secreted as inactive proenzymes from resting mast cells: alpha-protryptase and beta-protryptase (3–6). In contrast to alpha protryptase, beta protryptase is stored in its mature form in mast cell granules and released following mast cell degranulation as a teramer bound to heparin and chondroitin sulfate (3–6). Hence, the total MCT measured during anaphylaxis is predominantly of the beta tryptase isoform.

## Platform for Tryptase Assays

Globally there is only one manufacturer of MCT assays in the routine diagnostic setting. External quality assurance (EQA) schemes are used by laboratories as an assessment of accuracy. The only manufacturer of *in vitro* measurement of MCT represented in the UK based EQA scheme (UKNEQAS), is Thermo Fisher Scientific. The assay is available on a number of Thermo Fisher platforms, but all use the same methodology and reagents to measure total MCT (α and β isoforms). MCT is detected in serum using ImmunoCAP technology based on the principle of sandwich immunoassay. Anti-tryptase antibodies are covalently bound, to a cellulose derivative enclosed in a solid phase capsule. MCT present in serum will bind to the anti-tryptase in the solid phase. After a wash step to remove excess serum and unbound proteins, an enzyme-labeled secondary anti-tryptase antibody is added, once bound, will contain tryptase in a sandwich complex. After excess secondary antibody is washed off, the reaction is stopped. This chemical reaction causes a color change, fluorescence of which is measured and is directly proportional to the concentration of MCT in the serum sample.

## Performance Characteristics

### Analytical Performance

The manufacturer quoted assay imprecision, determined by inter and intra assay coefficient of variation (CV), across the full dynamic range of the assay, is consistent with that expected for the methodology (inter-assay CV ≤ 7% and intra-assay CV ≤ 4%) (7). This is supported by UK EQA data, where the overall CV for results per distribution (of >200 participants) consistently achieves a CV of <9.5% with an average CV of 7.3% over the last 6 distributions [personal communication; data only available to registered users of the UKNEQAS Tryptase scheme; https://www.immqas.org.uk/downloads/Participation_Handbook_2018_%202019_(V1).pdf].

## Time Kinetics of MCT

**Figure 1:**
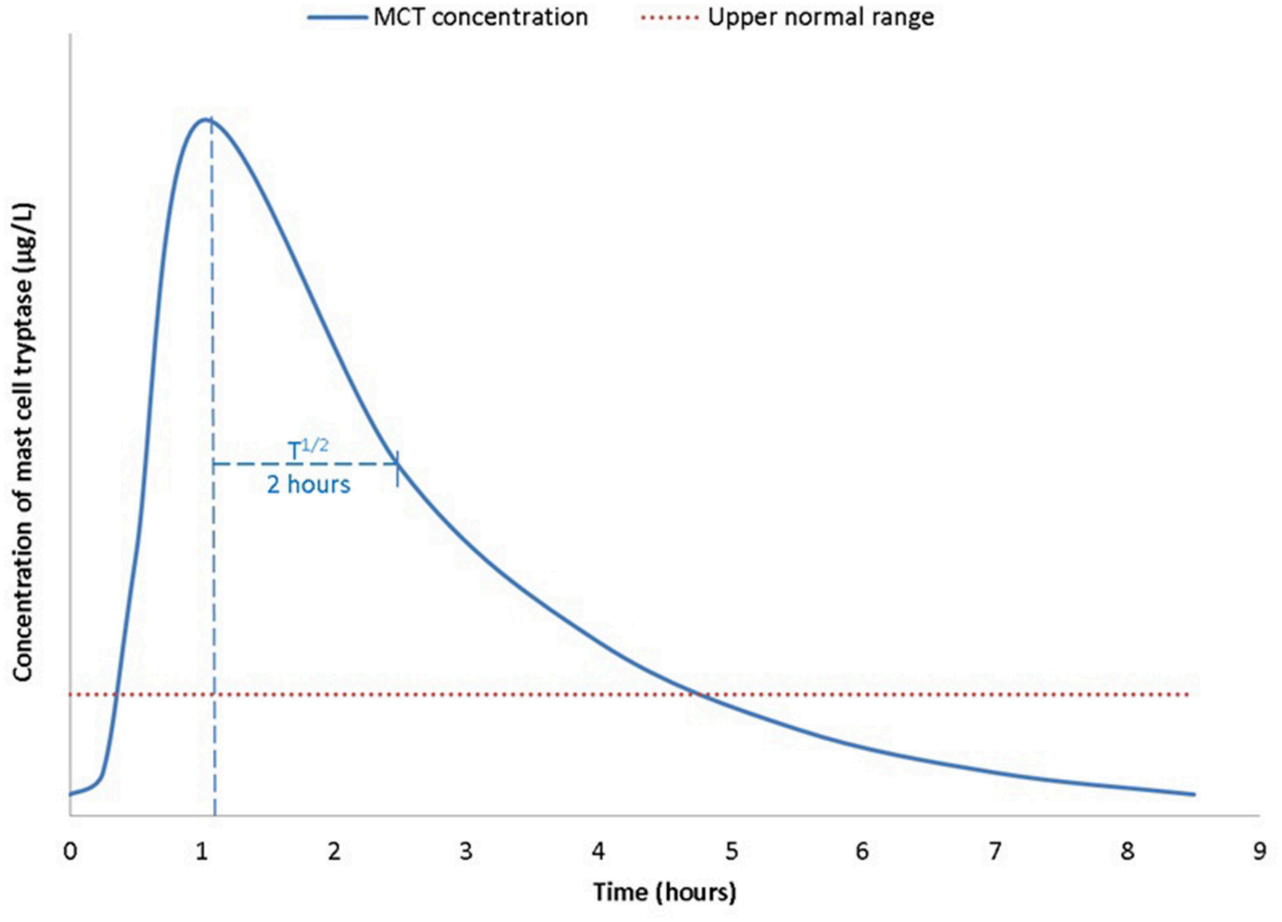
Time kinetics of mast cell tryptase (MCT) during an anaphylactic reaction, from a baseline concentration (pre-reaction), MCT is rapidly released from mast cells and peaks ~1–2 h post allergen exposure. T^1/2^ is ~2 h. Concentrations return to base line within 24 h after complete resolution of symptoms and signs of anaphylaxis.

At the onset of anaphylaxis, tryptase is released from mast cells alongside other mediators including cytokines, prostaglandin D2, and leukotriene C4 (3). Compared to histamine MCT has a longer plasma half-life of ~2 h (8) (Figure 1). Therefore, MCT has to date been the main target as a biomarker of anaphylaxis. Serum MCT concentration rises steeply in the first 90 min from the onset of reaction, followed by a steady decline in concentration following first order kinetics (8). It can take up to or beyond 24 h for levels to return to pre-reaction or baseline concentrations.

Serial samples must therefore be tested to demonstrate this natural progression and aid in the confirmation of anaphylaxis. The UK National Institute for Health Care Excellence (NICE), the British Society for Allergy and Clinical Immunology (BSACI) and the Association of Anesthetists of Great Britain and Ireland (AAGBI) guidelines recommend serial timed MCT samples–the first to be taken as soon as possible after emergency treatment has commenced, a second sample ideally within 1–2 h (but no later than 4 h) from the onset of symptoms and a baseline sample at ≥24 h (9, 10)^1^.

## Interpretation of Results

The manufacturer of MCT states a reference range of <11.0 μg/L (95 upper percentile). As with all laboratory tests, the test performance characteristics [sensitivity, specificity, positive predictive value (PPV), and negative predictive value (NPV)] will vary with patient populations due to differences in pre-test probability and the method of interpretation of data. Previous studies have reported performance characteristics based on a peak cut-off of the MCT measurement, a percentage change from baseline or a combination of these (11–13). We reported no significant difference in these two approaches in the context of peri-operative anaphylaxis based on area under the curve (14). Regardless, of the method of interpretation, the key determinant is to obtain one or more samples during an acute phase and within the half-life (<2 h) alongside a baseline measurement as described in the previous section. We validated the international consensus equation (15, 16) on mast cell activation (peak MCT >1.2 × baseline tryptase + 2 μg/L) in the context of peri-operative anaphylaxis and showed a high specificity and PPV for IgE mediated reactions (17).

An ideal laboratory test should have a high (>95%) specificity and PPV to confirm the condition, and a high (>95%) sensitivity and high NPV, to exclude and screen for a condition, respectively. This is difficult to achieve and the threshold is usually set depending on the purpose of performing the test, the clinical setting and patient population involved. Altering the cut off usually has a divergent effect on sensitivity and specificity. Therefore, increasing the cut-off for an acute MCT measurement or a percentage change in MCT, to enhance specificity will reduce sensitivity (12–14). Ideally, this should be performed by the respective laboratories offering the test, based on their local population and the process can be quite onerous and challenging for conditions such as anaphylaxis, since a large sample size will be required alongside controls for constructing receiver operating characteristic (ROC) curves to generate sensitivity, specificity, PPV and NPV.

## Practical Considerations Relating to MCT Requests in Clinical Practice

In specialist allergy practice, a diagnosis of anaphylaxis is based on clinical criteria as defined by the WAO in conjunction with an acute MCT, preferably with a baseline measurement (1, 2). A sample for acute MCT should be obtained within the half-life (~2 h) of tryptase. However, a very early sampling time (<30 min), may potentially generate a false negative result since it may not coincide with a peak rise. Whilst there is no international consensus regarding a specific time point within this narrow time window for obtaining an acute sample, the British guidelines^1^ recommend 2 sampling times (first as soon as possible and second with 1–2 h but no later than 4 h after onset of symptoms) to overcome false negative results. It is conceivable that 2 timed samples as per British guidelines may be challenging to comply with in a busy emergency department.

Studies undertaken by our group indicate a better uptake of national guidelines in the context of peri-operative anaphylaxis than in the emergency room. A recent UK wide National Audit Project 6 (NAP6) conducted by The Royal College of Anesthetists found that three timed samples were requested in 67% of cases, but only 45% met the BSACI guidelines for “immediate” sampling (<30 min) with 76% complying with sampling within the first hour (18). It is envisaged that the NAP6 has raised awareness amongst UK anesthetists regarding serial MCT measurements and accurate documentation of signs and symptoms in all cases of suspected peri-operative anaphylaxis.

We and others have reported that MCT measurements are not requested in a significant proportion of patients presenting to the emergency room with anaphylaxis, with compliance as low as 31–33% in retrospective studies rising only to 69% in a prospective study (13, 19, 20). Another challenging variable with respect to MCT measurement is that patients may not arrive in the emergency room within 2 h from the onset of the reaction, particularly in cases where symptoms worsen over a few hours (13, 21). A prospective study by Korosec et al. of patients with anaphylaxis presenting to the emergency room, found a median time to first MCT sample of 1 h 45 min, however the range was 1–31 h (21).

The poor rate of MCT requests from the emergency room may at least in part relate to the busy clinical environment with priority given to stabilizing the patient and lack of awareness amongst clinicians regarding MCT measurements. Also, the sampling time depends on the time taken by the patient to arrive in the emergency department which in turn may be influenced by the rapidity in the onset of symptoms and their severity.

Blood samples for MCT may be taken in EDTA, heparin or plain tubes without an anticoagulant and preferably analyzed within 5–7 days. MCT is stable *in vitro*. However, if there is going to be an anticipated delay in analysis, samples must be frozen at −20°C.

## Clinical Performance–Controlled Setting

Using manufacturer cut-offs, the specificity of an elevated MCT in a controlled clinical setting may show variations (Table 1). In a study involving insect sting challenges, Brown et al reported a relatively high specificity of 89% to 93% at a cut-off of 12 μg/L but sensitivity was poor (36%). However, ROC analysis found an optimal cut-off at 9 μg/L improved sensitivity to 55% with a marginal decline in specificity (87%) (22). This study however, involved a very small sample size. At a cut-off of 12 μg/L, Malinovsky et al. reported equal specificity in a prospective study in the setting of peri-operative anaphylaxis, but superior sensitivity (64%) (12). Similarly, Mertes et al. in the context of peri-operative anaphylaxis reported a sensitivity and specificity of 64 and 89%, respectively for a peak MCT of >25 mcg/l (11). In a similar setting, we reported a sensitivity and specificity of 64 and 74%, respectively at a peak MCT of >25 mcg/l (14).

**Table 1:** Performance of mast cell tryptase measurement in different clinical settings.

**Study**	**Sample number**	**Clinical setting**	**Causative agent**	**Number of samples tested per patient (time points, hours)**	**Cut-off**	**Sensitivity**	**Specificity**	**PPV**	**NPV**
**ACUTE/CONTROLLED SETTING**									
Brown et al. (22)	64	Prospective experimental Challenge–venom immunotherapy	Jack jumper ant	3 (Baseline–pre VIT, 0.25, 0.5)	9 μg/L	0.55	0.93	NS	NS
					12 μg/L	0.36	0.93	NS	NS
					Δ2 μg/L	0.73	0.98	NS	NS
Malinovsky et al. (12)	31	Prospective Routine clinical	General anesthesia	3 (0.5, 0.5–1, 24)	12 μg/L	0.64	1.00	1.00	0.53
					25 μg/L	0.41	1.00	1.00	0.41
					Ratio T_0_/T_24_ >3	0.63	0.83	0.92	0.42
Krishna et al. (14)	161	Retrospective Routine clinical	General anesthesia	3 (0–1, 1–2, 24)	25 μg/L	0.64	0.74	0.82	0.52
					33·6 μg/l	0.53	0.84	0.86	0.49
					Percentage change 506%	0.53	0.84	0.86	0.49
Baretto et al. (17)	82	Retrospective Routine clinical	General anesthesia	3 (0–1, 1–2, 24)	peak MCT >1.2xbaseline tryptase + 2μg/l	0.78	0.91	0.98	0.44
Dua et al. (23)	117	Prospective experimental Challenge - food	Peanut	3 (pre-challenge, 0, 1–2)	30% rise from baseline	0.53	0.85	NS	NS
**COMMUNITY**									
Buka et al. (13)	141	retrospective emergency	Various		12.4 μg/L	0.28	0.88	0.93	0.17
Korosec et al. (21)	31	Prospective Emergency	venoms (various)	2 (0 and 7/30 days)	11.4 μg/L	0.71	NS	NS	NS

*NS, not stated; PPV, positive predictive value; NPV, negative predictive value*.

Alternative methods of interpretations include a change in MCT concentration from baseline or “delta tryptase” (peak minus baseline) concentration may be more discriminatory than peak MCT alone. ROC analysis may determine a cut-off for optimal delta tryptase to improve the interpretation. For example, an increase in MCT of 2 μg/L in the study by Brown et al found a sensitivity of 0.73 (95% CI 0.39–0.94) and specificity 0.91 (95% CI 0.79–0.97), a superior performance to peak MCT alone (22). However, we were unable to demonstrate a significant advantage for a percentage change from baseline vs. absolute peak MCT measurement in the context of peri-operative anaphylaxis (14).

The relationship between peak and baseline MCT may also be expressed as a ratio between peak and baseline or between specific time point concentrations. For example, Malinovsky determined T_0_:T_24_ concentrations >3 μg/L as the discriminator. However, this was not found to be as good as increasing the cut-off of absolute MCT to 25 μg/L (12). As stated in the previous section, an international consensus equation has been recommended for the interpretation of mast cell activation (15, 16).

## Clinical Performance of MCT in Anaphylaxis Presenting to the Emergency Room

There are limited data available regarding the performance of MCT in an emergency room setting. Differences in performance of MCT amongst published studies may be attributable to multiple factors including definition of anaphylaxis employed (WAO diagnostic criteria were published in 2011), sample size, assays used for MCT measurement, sampling time, and etiology, and severity of anaphylaxis. We reported a sensitivity, specificity, PPV, and NPV of 28%, 88%, 0.93, and 0.17, respectively at a peak MCT of 12.4 μg/L (13). Practical considerations that are relevant to this setting also include awareness of the emergency room physicians regarding the role for MCT in anaphylaxis. Furthermore, a significant proportion of patients are discharged from the emergency room after a few hours following recovery, and may present to an allergy specialist at a different hospital or organization, and it may be challenging to coordinate the reports of acute MCT with a baseline measurement.

## Performance Variation with Culprit Allergen

**Figure 2:**
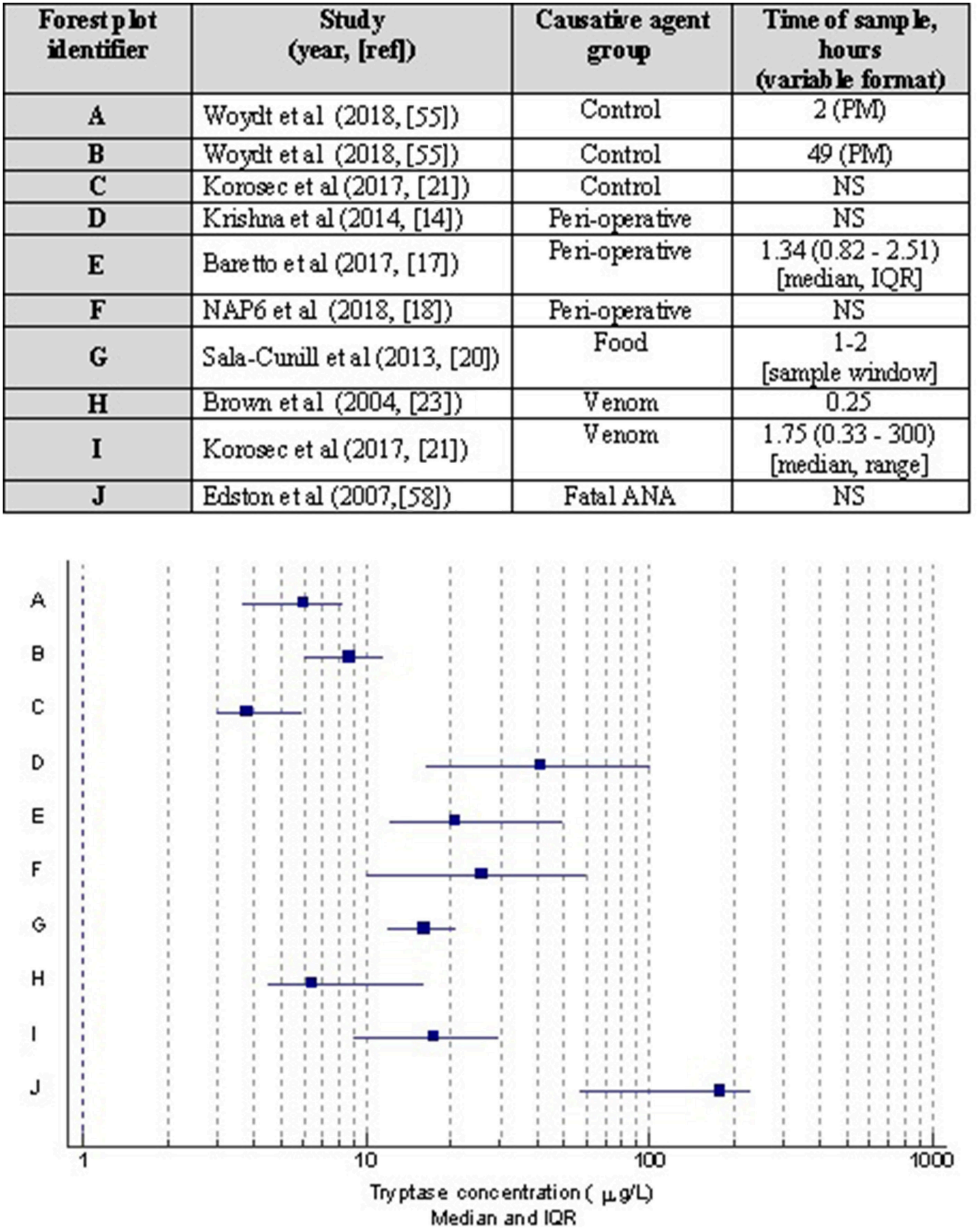
Forest plot showing median and interquartile ranges of acute MCT concentrations based on studies with different causative agents/settings of anaphylaxis. Corresponding table details the publication with 1st author, year, and reference; allergen inducing anaphylaxis, and MCT sampling time was taken for analysis. Note studies are limited to those where such data was presented. PM, post mortem; NS, not stated.

Regardless of the clinical setting, it is well-recognized that MCT may not differ from baseline levels in a significant number of cases of anaphylaxis (13, 14, 20, 24–26). This statement has to be cautiously interpreted since some of these studies were published prior to the publication of the WAO diagnostic criteria for anaphylaxis. Whilst, it is highly unlikely that this would have affected cases of severe anaphylaxis as graded by Brown et al. (27), some cases of mild-moderate anaphylaxis may not have made their way into the dataset in studies published before 2011, thus potentially affecting the performance characteristics of MCT. MCT levels have been shown to correlate with histamine. Furthermore, concurrent lack of elevation in MCT in some patients with a raised histamine highlights basophil involvement in anaphylaxis (26). Both histamine and MCT have been shown to correlate moderately with cutaneous symptoms of erythema and urticaria in anaphylaxis (26). There is some evidence that severity of anaphylaxis correlates with serum MCT and histamine (24, 25), with higher odds (13) for cases with hypotension likely to be associated with a raised MCT. These data have to be carefully interpreted since there was no standardization with respect to interpretation of MCT in anaphylaxis, i.e., cut offs for an absolute measurement or a percentage change. Although, it has been suggested that food-induced anaphylaxis is less likely to be associated with an increased MCT, we and others reported to the contrary (13, 19). In a recent study of double-blind-placebo controlled study involving peanut challenges, it was shown that at least a 30% rise in MCT was seen in 62.5% of cases (*n* = 150), out of which 9% developed anaphylaxis, giving a sensitivity and specificity of 53 and 85%, respectively (23). It is plausible, however, that the frequency and magnitude of elevation in MCT may be relatively lower in food-induced anaphylaxis as opposed to severe cardiovascular anaphylaxis seen in peri-operative setting (20) (Figure 2). This needs further investigation in prospective studies where the diagnostic criteria for anaphylaxis and methods of interpreting MCT are well-standardized (i.e., apply international consensus equation for mast cell activation (15, 16).

## The Clinical Relevance of a Raised Baseline MCT

There has been a great clinical interest in baseline MCT in the context of patients with a previous history of anaphylaxis. It has been suggested that a constitutively elevated baseline MCT (≥11.4 μg/L) significantly enhances the risk of severe anaphylaxis, as seen in patients with hymenoptera venom allergy (HVA) (28, 29). A cut off 20 μg/L constitutes one of four minor WHO criteria (30) for systemic mastocytosis, thus making it an important consideration for a referral for bone marrow studies. One large study in the context of HVA showed that the odds of severe anaphylaxis increases 5-fold between 5 and 15 μg/L of baseline MCT (31). Hence, the BSACI criteria for investigation and management of HVA emphasize on a baseline MCT measurement in all cases of systemic reactions to insect stings (32). A raised baseline MCT can also be seen in the context of chronic renal failure (33, 34) and hematological disorders (35) such as myelodysplastic syndrome, acute and chronic myeloid leukemia and chronic eosinophilic leukemia, and hence has to be carefully interpreted with relevant investigations for renal function, hematinics, and a clinical examination for mastocytosis and hematological malignancy. Furthermore, it has also been shown that 20% of the population may have an elevated baseline MCT due to hereditary alpha-tryptasaemia (autosomal dominant) due to increased germline copies of the alpha-tryptase gene (TPSAB1) (36). These patients have an elevated baseline MCT with or without non-specific multisystem symptoms. This has not yet been well-characterized.

## Use of Serum MCT in Forensic Medicine

A post mortem differential diagnosis of anaphylaxis can be a challenging task for a histopathologist. Whilst the diagnosis can be specifically investigated in suspected cases of anaphylaxis, it is also an important differential diagnosis of sudden death. The diagnosis is made considering a combination of variables including circumstances surrounding the death in conjunction with documented clinical findings by healthcare professionals, macroscopic features at necropsy (37) (cutaneous erythema/oedema, upper airway oedema, petechial hemorrhages, pulmonary congestion/oedema, mucus plugging of bronchi, and hyperinflation) and post mortem serum MCT measurements. Data regarding utility of post mortem MCT largely come from case studies and case series, the latter hampered by relatively small sample sizes. There are multiple variables that increase the uncertainty of measurement when serum samples are obtained from cadavers (38). These have to be carefully factored into the process of interpretation. Samples must be taken from femoral vein, and if not the source should be stated with the request to aid interpretation (38). Also, the method of sample acquisition should be noted, as femoral vein cut down could result in a slightly elevated level in comparison to aspiration with a needle and syringe (39). During autopsy procedures it may be easier to obtain blood samples from other locations. Cardiac chambers are known to have higher concentration of MCT compared to venous blood (38). This is likely to be due to passive diffusion of MCT post mortem from the relatively larger concentration of mast cells in the heart and lung. This may also be exacerbated by prolonged defibrillation or cardiac massage and sudden cardiovascular fatalities can also give rise to a raised MCT (38). Hemolysis occurs rapidly post mortem. High hemolysis affects most enzyme immunoassays (40) and in the case of MCT may cause falsely elevated results. Hemolysis may also indicate cell autolysis or tissue or blood vessel liquefaction. Cell lysis may cause increased MCT release. Hemodilution (caused from high fluid volumes ante-mortem) could also affect results causing false negative results, particularly if samples are taken from a central line. Therefore, post mortem samples must be taken from femoral veins.

A high post mortem MCT has also been reported in the absence of anaphylaxis. Asphyxia can contribute to a high MCT from femoral blood due to release from lung derived mast cells. Sudden infant death syndrome (SIDS) has also been associated with high MCT concentrations, although evidence regarding this is conflicting and the biological plausibility is unclear and there are no data on a direct cause-effect relationship (41–44). Deaths occurring from multiple trauma may have high MCT levels post mortem as opposed to deaths from single trauma or “non-trauma” deaths (45). Acute death after heroin injections has also been implicated in causing increased post mortem MCT. The time delay between death and sample acquisition can also affect post mortem MCT concentration. In a recent post mortem interval study by Woydt et al. (46), statistically significant differences were seen between paired samples at death and 3 h post mortem in a cohort where death was not due to anaphylaxis. One of the 20 cases breached the 44.3 μg/L cut off at 3 h. However, variation in MCT concentrations with post mortem intervals has inconsistent evidence.

Given the confounding factors discussed above and limitations of data, there is no standardized international reference range for post mortem MCT. Cut-off values proposed for forensic identification of fatal anaphylaxis vary between 10 and 54 μg/L (39, 47–49). It is worth noting that older publications (37) (prior to late 1990s) used β MCT as opposed to total (α and β) MCT in the last decade. Sun et al. recently published a systematic review and meta-analysis to address this question and concluded that at concentrations >30.4 μg/L, sensitivity and specificity for anaphylaxis were 68.5 and 83.9%, respectively (50).

At the present time, it is not a standard practice to obtain bone marrow samples during an autopsy. Given that fatal anaphylaxis is fortunately rare, this may be considered at least in cases where severe cardiovascular and/or refractory anaphylaxis has occurred since clonal mast cell disorders have been reported in such patients (51).

## Novel Biomarkers in Anaphylaxis

The recent identification of novel biomarkers that are either released from, or expressed on the surface of activated mast cells, eosinophils, and basophils have been aided by advances in cell purification, flow cytometry, and other laboratory techniques. There is growing evidence to support the investigation of other biomarkers, such as chymase, carboxypeptidase A3, dipeptidyl peptidase I (DPPI), basogranulin, CCL-2, and platelet activating factor (PAF) in the diagnosis of anaphylaxis. These are described below and summarised in Table 2.

**Table 2:** Summary of existing and new biomarkers in anaphylaxis, their (potential) limitations, strengths, and pharmacokinetics.

**Biomarker**	**(Potential) limitations**	**(Potential) strengths**	**Pharmacokinetics**
Mast cell tryptase (MCT)	Variable sensitivity influenced by clinical setting, timing of sample and set “cut offs”	Stable in serum, high specificity for type 1 hypersensitivity reactions (HSRs)	Half -life approximately 2 h
Histamine (8, 24, 25)	Short half-life, poor stability in serum *ex vivo*, Serum needs to be frozen promptly	High specificity for type 1 HSRs, concentration may correlate with reaction severity	<15 min
N-methyl histamine^*^	Measured in urine; may be increased in a variety of non-allergic conditions, and with bacterial contamination of urine sample. May be onerous for the patient as it involves 24 h collection.	Highly correlated with histamine in plasma	Methylhistamine is a stable end product of histamine
Chymase (52–54)	Limited data to verify	Predominantly found in mast cells, potentially stable in serum	Unknown half-life
Carboxypeptidase A3 (55, 56)	Limited data to verify.	Potentially detectable in serum and saliva; limited data suggests a rise in anaphylaxis where MCT is not elevated	Half-life longer than tryptase and this may potentially be an advantage if sampling time >2 h from onset of symptoms. Needs further research
CCL2 (21, 57)	Limited data to verify, Basophilic chemotactic factor	Uncertain, only preliminary data published. Serum levels may potentially correlate with the severity of anaphylaxis	Glycosylation influences its chemotactic potency and half-life *in vivo*
Dipeptidyl Peptidase I (DPPI) (54, 58)	Non-specific biomarker– expressed in many other cells	Uncertain, only preliminary data published	Unknown half-life
Basogranulin (59)	Limited data to verify	Uncertain, only preliminary data published. Unique secretory marker of basophils	Thought to have similar kinetics to histamine. Levels shown to be maximal at 15 min following stimulation with anti-IgE antibody
Platelet Activation Factor (PAF) (25, 60)	Short half-life and special sample requirements	PAF raised in anaphylaxis	Half-life <5 min

*Limited utility data is available for some; it is plausible that measurement of combined biomarkers will improve their diagnostic utility*.

* *https://www.immqas.org.uk/pru.asp?ID=316*.

## Chymase

In a similar fashion to tryptase, this serine protease is also largely found in the secretory granules of mast cells. One of the earliest reports of chymase as a potential biomarker for anaphylaxis examined 8 autopsy cases with anaphylaxis and 104 control cases without (52). Chymase was detected in all 8 autopsy cases with anaphylaxis, whilst it was only detected in 2 of the 104 control cases. This study also found a significant positive correlation between chymase and MCT levels in all 8 cases of fatal anaphylaxis. The authors also determined chymase to be relatively stable in serum, confirming its potential as a tool in the diagnosis of anaphylaxis. The same group more recently discovered positive chymase staining in lung mast cells from 3 autopsy cases of fatal anaphylaxis, in comparison to the control group of tissues associated with acute traumatic deaths (53).

Another study (54) established an enzyme immunoassay (EIA) to examine serum from patients with anaphylaxis provoked by food, drug and insect stings (*n* = 181), compared to a control group of healthy blood donors (*n* = 123) and patients with known allergies to food (*n* = 76) or drugs (*n* = 26). The authors found chymase levels to be greater in serum collected from patients within 8 h of anaphylaxis, compared to the control group (*p* = 0.0069). Furthermore, the concentration of chymase remained high at least 24 h after the onset of the reaction.

## Carboxypeptidase A3

Alongside MCT and chymase, carboxypeptidase A3 is another pre-formed chemical mediator of anaphylaxis released by activated mast cells.

Brown et al (55) measured carboxypeptidase A3 levels by EIA from blood and saliva, in patients undergoing allergy tests for suspected drug allergies (*n* = 33). The found carboxypeptidase A3 levels to be increased in saliva, but not serum, following a positive reaction to a drug challenge. Furthermore, baseline levels of carboxypeptidase A3 were higher in serum and saliva in patients who experienced symptoms on a drug challenge, compared to those who were asymptomatic. Baseline levels were also higher in patients who had historically suffered a severe reaction including cardiovascular and/or respiratory compromise, compared to those with only mild reactions.

Another study (56) also used a sandwich-based EIA to measure levels of carboxypeptidase A3 in cases of suspected anaphylaxis (*n* = 181), systemic mastocytosis (30), and control groups of healthy blood donors (*n* = 209), or individuals with bronchial asthma (*n* = 15). The authors found that carboxypeptidase A3 levels were significantly greater in the serum or plasma collected within 8 h of an onset of allergic reaction, compared to the control cohort. They also found carboxypeptidase A3 levels to be elevated (although range not stated) in 83% of anaphylaxis cases with an elevated MCT. Furthermore, carboxypeptidase A3 was also elevated in 70% of the 110 cases of suspected anaphylaxis cases that were MCT negative.

## CCL-2

In addition to mast cell mediators being used as biomarkers for anaphylaxis, there are promising data regarding major basophil chemotactic factor, chemokine (C-C motif) ligand 2, or CCL-2. Early studies have demonstrated an influx of basophils to inflammatory sites within several hours of allergen exposure, thus confirming a mechanism by which these cells might be recruited from circulation to the site of allergen exposure (61–63).

The potential use of CCL-2 as a biomarker in anaphylaxis was examined by one group (21), who measured CCL-2 at 3 time points (during the anaphylactic episode and in convalescent samples 7 and 30 days later) in 31 patients presenting to the emergency department with anaphylaxis. The authors found that the absolute numbers of circulating basophils were significantly lower during reactions, compared with both the samples taken in convalescence and in a healthy control cohort. The study also found the concentration of CCL-2 to be significantly higher in anaphylaxis, compared to both the samples taken in convalescence and in the healthy control cohort. They measured CCL-2 levels in a selection of peanut allergy patients undergoing a double-blind placebo-controlled peanut challenge. The results showed CCL-2 levels increased significantly at the time of objective symptoms, compared with baseline. Furthermore, the rate of CCL-2 increase was significantly greater in the active food challenge group compared to the placebo control. The concentration of CCL-2 was also noted to return to baseline within 2 h of symptom onset. The authors concluded that at a cut-off level >334 pg/μL, the estimated sensitivity and specificity for CCL-2 measurements were 94 and 96%, respectively.

In another more recent study (57), CCL-2 was measured in serial serum samples from 60 patients with differing severity of systemic allergic reaction (Mueller grades I–IV) and compared to 98 healthy controls. The study found CCL-2 levels to be significantly higher in the patient group, compared to the healthy control cohort. The authors reported CCL-2 levels to be significantly higher in acute serum samples from patients with grade III and IV severity reactions, than in those patients with systemic grade I reaction. Using paired baseline measurements of CCL-2, they also showed a significantly higher increase of CCL-2 in patients with grade IV anaphylaxis compared to those with grade I reaction. This study not only highlights the use of CCL-2 as a biomarker for anaphylaxis, but also suggests CCL-2 levels in acute serum correlate with the severity of the reaction.

## Dipeptidyl Peptidase I (DPPI)

DPP1, also known as Cathepsin C is a member of the papain family of proteases and is expressed by numerous cell types including both mast cells and basophils. Studies using DPP1 knockout mice have proposed a role in activation of chymase, but not tryptase (58). This is supported by investigation of DPP1 as a useful serum marker of anaphylaxis (54), where elevated chymase concentrations in patients with anaphylaxis also correlated with levels of DPP1, but not tryptase.

## Basogranulin

The novel basophil granule protein, termed “basogranulin” has been shown to be released by basophils in parallel with histamine and is thought to be dependent on the same receptors and signaling pathways as those for histamine release (59). Further work is needed to assess the extent and role of basophil activation in anaphylaxis and whether basogranulin could be used as a suitable serum marker.

## Platelet Activation Factor (PAF)

There has been an interest in measuring PAF and PAF acetylhydrolase (PAF AH), an enzyme inactivating PAF. Platelet activation factor is a proinflammatory phospholipid secreted by mast cells, monocytes, and tissue macrophages (25). PAF binds to its receptors on platelets, monocytes, macrophages, and neutrophils. Vadas et al. (60) reported an elevated PAF concentration in anaphylaxis which correlated with severity. There was an inverse correlation between PAF and PAF AH activity. They also measured PAF AH activity in patients with fatal anaphylaxis in peanut allergy and compared it with controls–PAF AH was significantly reduced in fatal anaphylaxis samples. In another study, Vadas et al. showed that PAF correlated better than MCT and histamine with severity of anaphylaxis (25). One of the challenges with measurement of PAF and PAF AH in a routine clinical setting is its very short half-life and special sampling and transport precautions that are required thus making it an unattractive candidate for routine use.

## Future Perspectives and Conclusions

Anaphylaxis remains a clinical diagnosis and biomarkers have no role in acute management. However, they have an important place during specialist allergy evaluation to confirm the diagnosis and distinguish anaphylaxis from its mimics such as severe asthma, vocal cord dysfunction, factitious disorder or somatoform disorders, hypotensive crisis due to non-allergic causes and in certain circumstances where there might be a paucity of information relating to the index episode. At the present time, the gold standard laboratory test is MCT, although other biomarkers such as chymase, carboxypeptidase A3, and CCL-2 hold promise for the future, providing sample requirements, assay platform, process, and costs are compatible for routine use in clinical diagnostic laboratories. Prospective well-designed multicenter studies applying the WAO diagnostic criteria for anaphylaxis on well-characterized patients with serial timed blood samples and standardized methods of interpretation of serum MCT are needed to firm up on the sensitivity, specificity, PPV and NPV. There may be a role for a combination of MCT and a newer biomarker/s to enhance sensitivity and specificity. Multiplex approach may improve efficiency in a high throughput diagnostic laboratory using a relatively small amount of serum. Validation of biomarkers in saliva might be useful in the interpretation of food challenges, particularly those with subjective symptoms and minimal or no objective signs.

One of the most intriguing aspects of human anaphylaxis is that some patients with severe symptoms do not show an elevated MCT. This raises the possible involvement of other *hitherto* unidentified biomarkers and possibly unidentified pathways. A new classification system based on phenotypes, endotypes, and biomarkers has been proposed that might enable a more robust stratification of patients for appropriate investigations and selection for desensitization, in particular those developing adverse reactions to biologics, anti-cancer drugs and radiocontrast media (64). The systems biology and proteomics approaches applied on serum obtained during an acute phase and convalescence may be worth exploring. Greater understanding of the pathogenesis underpinning anaphylaxis might pave the way for better risk stratification of patients, improve diagnostics and create opportunities to develop novel immune-based treatments such as venom immunotherapy.

## Author Contributions

SB, TW, and MK provided substantial contributions to the conception, design, content, and drafting of the manuscript. AH, RJB, and RLB provided critical appraisal of the manuscript as well as substantial editorial contributions.

### Conflict of Interest Statement

The authors declare that the research was conducted in the absence of any commercial or financial relationships that could be construed as a potential conflict of interest.

## Footnotes

^1^http://guidance.nice.org.uk/CG134
